# Informing evaluation of a smartphone application for people with acquired brain injury: a stakeholder engagement study

**DOI:** 10.1186/s12911-018-0611-0

**Published:** 2018-05-30

**Authors:** Jade Kettlewell, Julie Phillips, Kate Radford, Roshan dasNair

**Affiliations:** 1 Division of Rehabilitation and Ageing, School of Medicine, University of Nottingham, D1409, Medical School, Queens Medical Centre, Nottingham, NG7 2UH UK; 2 Division of Rehabilitation and Ageing, School of Medicine, University of Nottingham, B114, Medical School, Queens Medical Centre, Nottingham, NG7 2UH UK; 3 Rehabilitation Research (Long Term Conditions), Division of Rehabilitation and Ageing, School of Medicine, University of Nottingham, B102, Medical School, Queens Medical Centre, Nottingham, NG7 2UH UK; 4 Clinical Psychology & Neuropsychology, Division of Psychiatry & Applied Psychology, C22, Institute of Mental Health, Jubilee Campus, University of Nottingham, Nottingham, NG7 2TU UK

**Keywords:** Brain injuries, Technology, Smartphone application, mHealth, Self-monitoring, Engagement study, Behaviour change

## Abstract

**Background:**

Brain in Hand is a smartphone application (app) that allows users to create structured diaries with problems and solutions, attach reminders, record task completion and has a symptom monitoring system. Brain in Hand was designed to support people with psychological problems, and encourage behaviour monitoring and change. The aim of this paper is to describe the process of exploring the barriers and enablers for the uptake and use of Brain in Hand in clinical practice, identify potential adaptations of the app for use with people with acquired brain injury (ABI), and determine whether the behaviour change wheel can be used as a model for engagement.

**Methods:**

We identified stakeholders: ABI survivors and carers, National Health Service and private healthcare professionals, and engaged with them via focus groups, conference presentations, small group discussions, and through questionnaires. The results were evaluated using the behaviour change wheel and descriptive statistics of questionnaire responses.

**Results:**

We engaged with 20 ABI survivors, 5 carers, 25 professionals, 41 questionnaires were completed by stakeholders. Comments made during group discussions were supported by questionnaire results. Enablers included smartphone competency (capability), personalisation of app (opportunity), and identifying perceived need (motivation). Barriers included a physical and cognitive inability to use smartphone (capability), potential cost and reliability of technology (opportunity), and no desire to use technology or change from existing strategies (motivation). The stakeholders identified potential uses and changes to the app, which were not easily mapped onto the behaviour change wheel, e.g. monitoring fatigue levels, method of logging task completion, and editing the diary on their smartphone.

**Conclusions:**

The study identified that both ABI survivors and therapists could see a use for Brain in Hand, but wanted users to be able to personalise it themselves to address individual user needs, e.g. monitoring activity levels. The behaviour change wheel is a useful tool when designing and evaluating engagement activities as it addresses most aspects of implementation, however additional categories may be needed to explore the specific features of assistive technology interventions, e.g. technical functions.

**Electronic supplementary material:**

The online version of this article (10.1186/s12911-018-0611-0) contains supplementary material, which is available to authorized users.

## Background

### Acquired brain injury

Acquired brain injury (ABI) is defined as damage to the brain that has sudden onset and occurs after birth [1]. It includes both traumatic head injures (e.g. road accidents, falls) and non-traumatic causes such as vascular incidents (e.g. stroke), infections (e.g. meningitis), brain tumours hypoxic injuries and other non-generative neurological conditions [1]. Approximately 956 people were admitted to hospital every day due to ABI in 2013–14, which has increased by 10% since 2005–06 [2]. Approximately 1.3 million people in the UK are living with a traumatic brain injury [3], so the number of people with ABI will be even larger. ABI results in a myriad of persistent deficits in cognition (difficulties with memory, problem solving, planning, slower information processing, etc.), psychological and behavioural problems (e.g. increased anxiety, irritability), physical problems (epilepsy, fatigue, etc.), and social problems (e.g. isolation, reliance on family members). The main objective of rehabilitation with people with ABI is to help them recognise their problems, and find strategies to help them achieve the highest quality of life.

### Assistive technology

Assistive technology (AT) is an umbrella term that refers to devices developed for people with disabilities, which can be assistive, adaptive, and rehabilitative. AT aims to compensate for loss of function and promotes greater independence, by providing individuals with the support they need to carry out tasks and move towards leading more fulfilling lives. In this study, we focus on ATs for cognition, which refers to technologies that may enhance, enable or extend cognitive function when used. This type of AT does not aim to restore cognitive function, but is used as a compensatory device to support these functions, for example, memory, executive function, language, and attention. Although ATs exist to support rehabilitation, there is limited technology available to support self-management and functional outcomes specifically for people with ABI [4, 5]. With such a large section of the population recovering from ABI, many in their youth, there is a need for ATs that can support all aspects of rehabilitation.

We identified several ATs in the literature available for ABI survivors and a recognised benefit of reminders to improve prospective memory and promote independence [4, 6–10]. ‘Neuropage’ is a well-established paging device that provides scheduled reminders for people with cognitive problems [9, 11–13]. Multiple studies have corroborated the value of reminder devices like Neuropage, electronic calendars, and personal digital assistants (PDAs) for ABI survivors, in improving performance of everyday tasks [10, 14–17].

Mobile health (mHealth) is becoming increasingly popular and applicable to healthcare. The World Health Organisation (WHO) defines mHealth as, medical or public health practice supported by mobile devices, such as mobile phones, PDAs, pagers and tablets [18]. With smartphone ownership in the UK at an estimated 76% of the adult population, mobile technology is becoming an integral part of everyday living [18, 19]. Smartphone applications (apps) for people with ABI (e.g. Evernote, Google Calendar, Family Tracker, etc.) usually have a calendar or reminder function. However, few apps have been systematically tested in this population, with the majority evaluating the efficacy of such technologies with studies with small sample sizes [7, 15, 20, 21]. One study explored the barriers to technology use, with a smartphone app called ‘ForgetMeNot’, which incorporates unsolicited prompts to encourage users to input more reminders [22]. Another study explored the use of a mHealth app (PEAT), designed as a scheduling assistant, but was found to be only as effective as paper-based methods [8].

Given the ubiquity of mobile phones and an increasing availability of mHealth apps, we expect that these technologies will be adopted more within healthcare services [23, 24]. Despite a growing awareness of mHealth, there is, however, limited research exploring the benefit of these technologies on executive function such as self-monitoring, problem solving, social interaction, initiation of tasks, and volition [4, 5, 25]. Other common co-morbidities of ABI such as fatigue, depression, anger, anxiety, and lack of confidence, also affect independence and social participation. These sequalae or ‘invisible’ symptoms are among the greatest unmet rehabilitation needs for this population [26–29]. There is an urgent need for mHealth apps to support people carry out these functions following ABI, and to promote independence and autonomy.

### Problems with implementation

Many studies focus on the effectiveness of mHealth interventions, specifically reminding technologies [7, 9, 30, 31], however few address the barriers to use - essential to the development and implementation of these technologies [5, 22, 32–34]. It is important to consider the potential use of the technology in clinical practice when designing and evaluating mHealth interventions. Implementation studies attempt to identify the key features of an intervention and consider its applicability in practice, to better inform necessary developments and meet the needs of the target population [22, 34]. A report by the Voluntary Organisations Disability Group (VODG) identified problem areas associated with implementing smart technologies, such as a lack of care pathway commissioning, a lack of public awareness of mobile technology, and the unchangeable (and sometimes unhelpful) mind-set of healthcare professionals (HCPs) [35]. Some healthcare providers who regard themselves as traditional ‘caregivers’, might have concerns that physical care and social care may be lost to the increasing use of technology [35, 36]. There may be a certain level of reluctance to change, especially if intervention uptake has the potential to result in the loss of (their) jobs and reduced clinical support. Potential barriers to the uptake of mHealth may be commissioning of clinical services, technical issues (e.g. poor internet connection), fear of change, lack of leadership, and risk of investment [36]. However, increasing staff efficiency, ability to provide information faster, potential cost savings, and increased patient dignity, control and independence may be potential enablers to the uptake of technology [36].

### Brain in Hand

A new mHealth app called ‘Brain in Hand’ (BiH) is currently being implemented in various settings for autism and mental health. BiH is a web-based software that synchronises with a smartphone app. It is readily available and simple to use. The software helps users create a structured daily routine for difficult to remember tasks or problem situations. The smartphone app gives patients instant solutions, which can be easily accessed in real time, thus allowing them to manage their distress levels and improve independence. BiH has a reminder function that enables patients to set alarms for important appointments and tasks, with the ability to record task completion. BiH also has an online portal, which comprises a diary and timeline. The portal acts as a monitoring system whereby a user, carer, mentor or HCP can track app usage. The timeline can record functions selected on the app, whether it be a solution or completion of task.

App data are recorded in real time and stored in the ‘cloud’, allowing users and HCPs to monitor and better understand issues that cause distress. A traffic light alarm system, prompts users to record their anxiety levels at a specific time: red (I am not coping), amber (I am struggling, but I am coping) and green (I am coping). There is also the option to use this function to access telephone support if needed i.e., when the red button is pressed.

### Behaviour change wheel

**Fig. 1:**
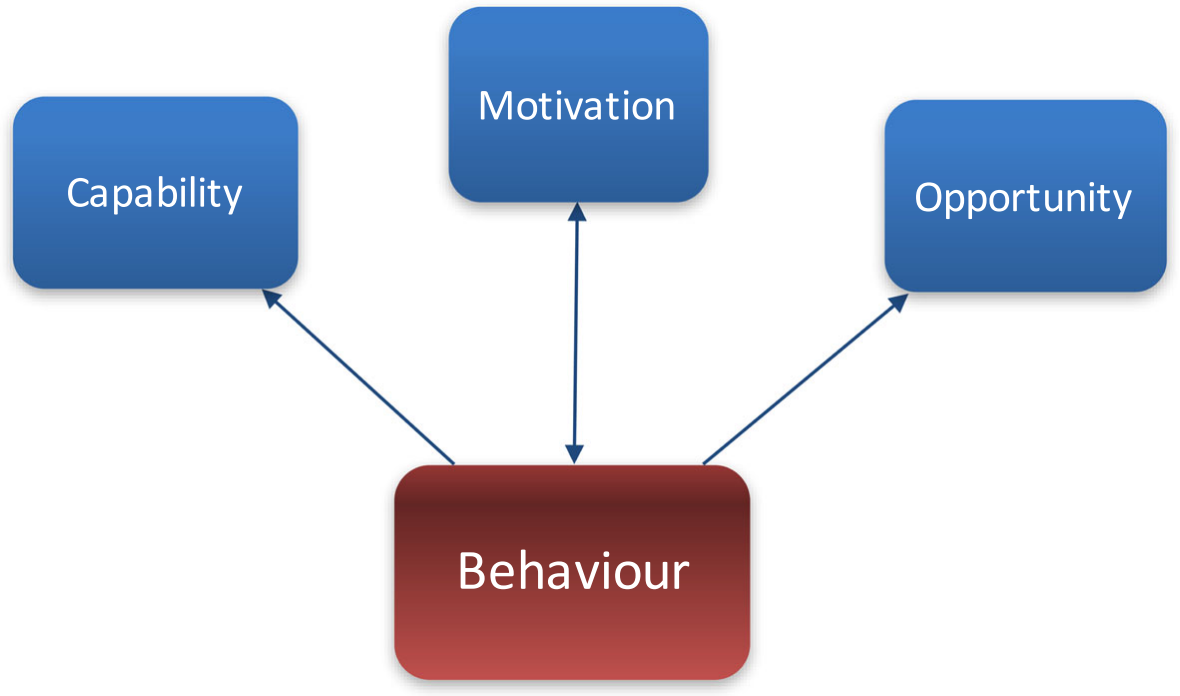
Model of Behaviour Change (COM-B) [38, 45]. The relationship between capability, opportunity, motivation and behaviour, forming the basis of the BCW. The arrows demonstrate how each part in the COM-B model interacts within the behaviour system. Capability influences motivation, opportunity also influences motivation, which then impacts on behaviour. Motivation tends to drive behaviour, however behaviour can influence motivation. Capability moderates the link between behaviour and motivation, as does opportunity. The behaviour system exists as a cycle and is an important model to consider when designing and evaluating interventions that aim to change behaviour

We chose the Behaviour Change Wheel (BCW) to inform the method of this study, as the technology (BiH) being evaluated aims to encourage changes in behaviour [37]. The BCW model suggests that changing behaviour is based on three components: capability, opportunity and motivation (COM-B). Capability refers to an individual’s psychological and physical capability to engage in an activity, opportunity refers to factors not directly linked to the individual that make that behaviour possible (e.g. having access to a smartphone), and motivation refers to reflexive and emotional responses that make the behaviour possible (e.g. knowing something will work) (see Fig. 1). [37, 38].

Examples of anticipated behaviours when using BiH include: activity and medication reminders, fatigue and anxiety monitoring, reminder of strategies to use when a problem occurs. The BCW was appropriate for this study because it considers other factors that might influence use, such as the need for training to use the technology. Although the BCW model considers factors such as environmental restructuring (i.e., changing the physical context and enablement), increasing or reducing barriers to use the app, discussions with experts in the use of BiH felt this did not fully explore the usability and acceptability of the actual technology. Therefore, we identified studies that had evaluated technology and used feedback questionnaires to inform potential views of the app.

### Rationale

A literature review suggested there is a need to better understand the use of technology in health, and various studies have identified potential problems with the uptake of mobile technologies, such as adaptability, cost, compatibility with existing systems and complexity [35, 36, 39]. The research literature highlights the importance of identifying barriers through consultation with key stakeholders and collaboration, early in the implementation process [39–41]. The rationale for this study was therefore to evaluate the usability and acceptability of the smart technology, and explore its potential use in ABI rehabilitation, through early stakeholder consultation.

### Aims

The aim of this paper is to describe the process undertaken to:

- Explore the barriers and enablers, and the potential use of BiH technology in clinical practice with ABI survivors.
- Identify possible adaptations of the technology to meet the needs of the ABI population.
- Determine if the BCW could be used as model for stakeholder engagement.

## Method

**Fig. 2:**
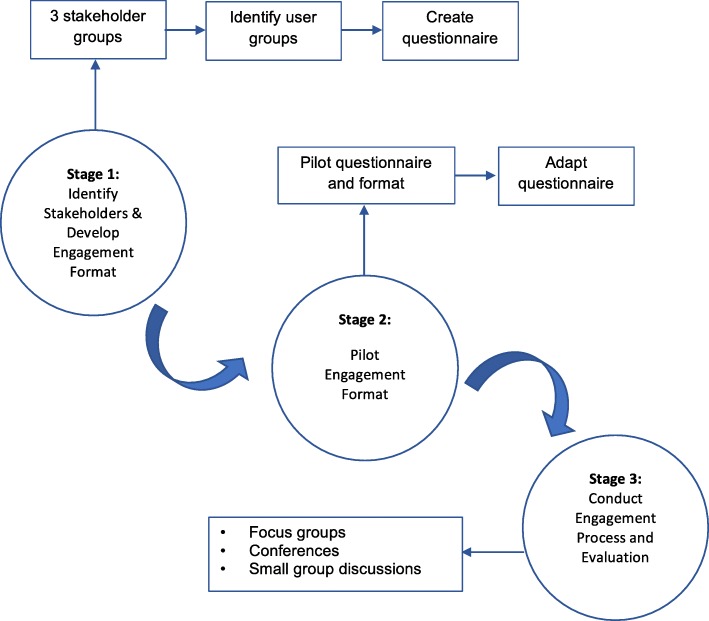
Summary of engagement method. The iterative method used to identify stakeholders and carry out engagement activities

As the aim of BiH technology is to help users change their behaviour, the method of engagement was informed by the BCW model [37]. Engagement was defined as consulting with patients, carers and HCPs to obtain their views about the technology through different activities. This consisted of verbal engagement with the research team, focus groups, meetings with users and professional groups at their workplace, conferences and completion of questionnaires. We considered a mixed method approach appropriate for this type of study. We followed Johnson, Onwuegbuzie and Turner’s view [42] of taking a broad interpretation of the word ‘methods’ here, in that it incorporates a variety of data collection methods, research approaches, and philosophies. We felt that this approach would enable us to identify various perspectives on the potential uses, enablers and barriers of the technology, and enable us to evaluate the potential use of the BCW as a tool for engagement, by using both quantitative and qualitative methods. We also felt that a mixed methods approach would increase the breadth of the research, by providing a fuller picture of people’s experiences, attitudes and feelings about the technology. The data collection process was divided into three stages, with an ongoing iterative approach (Fig. 2). We followed the UK Health Research Authority (HRA) guidance regarding ethics approval via an online assessment tool, and were advised that it specific ethical approval was not required, as this project was viewed as stakeholder consultation. Participants were identified through voluntary organisations, special interest groups and conferences that had a focus on ABI/technology, and at organised events for the lay public.

### Stage 1: Identify stakeholders and develop a format for engagement

The study management group consisted of academics and clinicians. They identified the stakeholder groups as: ABI survivors who would use the technology, carers or support workers who would mentor people with ABI and support them to use BiH, and HCPs who would potentially recommend using BiH as part of a treatment programme. We took a pragmatic approach to accessing the identified user groups, and incorporated engagement activities within meetings held by stakeholders. Additionally, focus groups were held to specifically engage both ABI survivors and carers.

As BiH is a novel mHealth app and currently unused with the ABI population, we provided potential stakeholders with information about the system during small groups or presentations, during which we explained the functions of BiH, its potential uses in daily life and in clinical practice. In addition, we gave people the opportunity to explore and experience the app themselves using a smartphone or iPad.

Our academic advisory panel suggested using existing technology questionnaires such as the Mobile Application Rating Scale (MARS) [43] and the System Usability Scale (SUS) [44] to capture stakeholders’ opinions about similar technologies. These scales were evaluated by the researchers and the most appropriate elements incorporated into a questionnaire. The study management group agreed that a questionnaire would provide further in-depth quantitative data (particularly relating to the technical aspects of the app), and give people the opportunity to provide feedback outside of a group setting. A questionnaire was designed to be piloted with all stakeholders.

### Stage 2: Piloting the engagement format

To determine if our methods of engagement were appropriate, and would obtain the relevant feedback, we piloted the format of discussion and questionnaire with a small group (ABI survivors, carers and HCPs) that had an interest and familiarity with smart technology. Feedback obtained from this activity informed necessary changes to the engagement approach and questionnaire. We contacted the local technology special interest group known to the researchers and a meeting was arranged. Participants were recruited during the group session and questionnaires completed following discussion. The group is run by a local therapist involving people with an ABI who have an interest in and review technology for, and on behalf of people with ABIs. Group members are former service users/carers themselves and interested in helping other people with an ABI. The group was chosen because of its focus on technology. Participants were not current UK health department service users.

### Stage 3: The engagement process

The final stage involved identifying opportunities to engage with the stakeholders. We identified conferences and meetings where we could present and discuss the technology with professionals. We were invited to present our study and demonstrate the technology at a head injury conference organised by the local brain injury community service, where most of these participants were recruited. This was held in close proximity to the university and comprised a range of professionals (therapists, solicitors, case managers and business owners). We informed the professionals that we were soliciting feedback from them as stakeholders to help us identify the potential for the technology to help people with ABIs achieve their rehabilitation goals (and inform outcome measurement for a subsequent study). Professionals were invited to complete questionnaires following discussion if they wished (Additional file 1). Further participants were recruited at meetings identified by the authors (JK and JP) through their existing professional contacts, which all had a focus on ABI and/or technology. These groups exist to share information about specific aspects of their areas of interest, and to offer support, guidance, and feedback for various projects or cases. As before, stakeholders were invited to complete questionnaires following the meeting.

To ensure ABI survivors and carers were fully involved, we organised focus groups. The APEASE criteria [38, 45] along with the BCW was used to create a topic guide for the focus groups. The focus groups were facilitated by two people (JK, a PhD researcher, and JP, a research/clinical occupational therapist). The group discussions were audio recorded, analysed by the groups facilitators, and the findings discussed with the study management group. Views generated in the focus groups and from other engagement activities were mapped onto the BCW behaviour components.

The two focus groups comprised 8–10 service users and carers in each group. ABI participants and carers were recruited from various sources. Some participants were identified using a database of people with ABIs (who had previously expressed an interest in taking part in brain injury research) at the University of Nottingham. These people had previously consented to being contacted by researchers in our department regarding future studies and being involved in patient and public involvement (PPI) activities. This database is stored securely within the University and accessible only to members of our research team (long term conditions). It was accessed by author JP and potential participants were invited to take part in a focus group by email, giving them the opportunity to respond in their own time. Other ABI participants and carers were identified via social media posts by Headway and the Stroke Association (two UK charities), from an existing stroke specific PPI group (University of Nottingham Stroke Partnership Research Group (UNSPRG)), and local Headway and stroke groups. We used purposive sampling to ensure a heterogeneous sample, including people who did and did not use technologies.

## Results

### Stage 1: Identify stakeholders and develop a format for engagement

We engaged with 50 stakeholders: 20 ABI survivors (16 completed the questionnaire), 5 carers (4 completed the questionnaire), and 25 professionals (21 completed the questionnaire) who worked with people with ABI including therapists from NHS and private sector, and solicitors. Demographic characteristics of the stakeholders are presented in Table 1.

**Table 1:** Demographic information of stakeholders

Total = 50		Age			Gender	
		18–30	31–50	51+	Male	Female
ABI survivors (*n* = 20)	n	3	9	8	15	5
	*% total*	*6%*	*18%*	*16%*	*30%*	*10%*
Carers (*n* = 5)	n	1	0	4	2	3
	*% total*	*2%*	*0%*	*8%*	*4%*	*6%*
Professionals (*n* = 25)	n	2	12	11	4	21
	*% total*	*4%*	*24%*	*22%*	*8%*	*42%*
Overall	n	6	21	23	21	29
	*% total*	*12%*	*42%*	*46%*	*42%*	*58%*

### Stage 2: Piloting the engagement format

We piloted our engagement format with an ABI user group (*n* = 6) that had a special interest in smartphone apps and were familiar with technology like BiH. Feedback from this stakeholder group suggested ideas for technological development and identified barriers to uptake, thus indicating that the format did facilitate informed discussion of the smartphone app. Based on their feedback, we adapted the questionnaire to make it more accessible and easier to complete by creating two versions: one for ABI survivors and carers (Additional file 2) and one for professionals (Additional file 1). We also removed the frequently unanswered and irrelevant questions that did not provide useful information about BiH.

### Stage 3: The engagement process

We took a pragmatic and opportunistic approach to consult with the stakeholders. All were asked to complete a questionnaire. The engagement activities included:

- A formal presentation followed by a discussion with 30 professionals (therapists, case managers, solicitors) at an ABI meeting.
- Three informal presentations, to discuss and demonstrate BiH to two groups of therapists treating neurological patients (six per group), an ABI technology user group, and at a day centre (Headway) specifically for people with brain injury.
- An exhibition stand at a lay stroke conference where information was provided to delegates (stroke survivors, carers and professionals) on a 1:1 basis. The environment was not suitable for questionnaire completion. Some delegates took questionnaires home, but none were returned. Suggestions for improvement and any comments about the technology were noted on the day.

### Results from discussion and questionnaires

The main points identified by the focus groups and other discussions complemented the questionnaire responses. However, the questionnaire approach limited responses, and did not allow individuals to elaborate on their views. For example, during one focus group, a suggestion was to change the word ‘problems’ to ‘issues’ on the app, as the ABI survivor felt the language was negative. Another person suggested linking the app to activity monitoring. This information would not have been captured with questionnaires alone. However, the questionnaire enabled us to quantify some responses such as potential uses and reporting on the technical aspects of the app (e.g. appearance of BiH). Over half of stakeholders stated that seeing the app made them want to use it (65%, 17/25). This suggests that the app is visually appealing.

### Capability

Participants felt they would be confident using the app if they were existing technology users (22/24, 95%), but only a quarter of people felt it was completely appropriate for the ABI population (6/25, 24%). This finding is reflected by comments from professionals who felt that users needed an awareness of their problems and an ability to use strategies for the app to be effective (Table 2). Most stakeholders identified clear barriers during group discussions: visual and physical impairments, lack of technical skill, poor literacy skills, and cognitive problems faced by ABI survivors. Professionals identified potential problems associated with the use of BiH, such as difficulty switching between screens.

**Table 2:** Summary of enablers and barriers identified by stakeholders mapped onto BCW behaviour components

	Enablers	ABI	Carers	Professionals		Barriers	ABI	Carers	Professionals
Capability	Physical ability to use smartphone	●	●	●	Capability	Visual and physical impairment	●	●	●
	Competent at using smartphone	●	●	●		Lack of technical skill	●		●
	Awareness of their problems		●	●		Poor literacy skills			●
	Able to use strategies	●		●		Cognitive problems e.g. switching between screens	●		●
Opportunity	Have a smartphone	●	●	●	Opportunity	Cost and competition from other apps	●	●	●
	Having the opportunity to personalise use	●	●	●		Losing smartphone and battery problems	●		
	Enables users to record symptoms as they happen	●		●		Technology not reliable e.g. loss of information if phone updated	●	●	
	Computerised record of use (e.g. to track goal attainment)			●		Incapability with existing systems i.e. will it sync with computer diary	●		●
	Offers opportunity to self-monitor and alter use	●		●		Work place restriction not allowing use	●		●
Motivation	Using a phone seen as ‘normal’ behaviour i.e. reduced stigma	●			Motivation	No interest in the use of technology or existing systems in place e.g. paper diary	●	●	●
	Potential to be personalised to individual needs	●	●	●		Do not see a use for it	●	●	●
	Motivated if they can see a use for it e.g. problem and solution system	●	●	●		Feeling the app is too complicated for them to use or learn to use			●
	Feeling confident to use the app	●				Loss of motivation and disengagement with rehabilitation process			●
	May allow the user to feel like they are taking control of their life again		●	●		Feeling as though they are being monitored	●		
	Do not want to use paper diary	●				Too time consuming to set up/edit	●	●	●
						Too many reminders may be annoying	●	●	●
						Lack of existing evidence/use in the ABI population	●	●	●

Summary of the barriers and enablers identified by ABI survivors, carers and professionals mapped onto the behaviour components of the BCW. The marked boxes indicate whether that stakeholder identified or discussed that barrier or enabler.

### Opportunity

All stakeholders identified the need to have a smartphone as one of the most important enablers and any potential cost of the app as a significant barrier. Only 24% (9/37) said they would be willing to pay for the app, with a further 54% saying they would consider paying for the app depending on the price (Table 3). The groups identified a potential barrier to uptake being competition from apps that have similar functions, but are free or have a one-off cost. Technological problems were identified as major barriers, specifically by individuals who were worried about loss of phones, the battery running down, compatibility with other systems and loss of data following phone updates. There was a concern about use in the work place, as for many people it is not appropriate to use a phone whilst working. With regards length of use, 49% (20/32) of stakeholders thought the app would be used by someone with ABI for more than 12 months and only 15% (6/32) felt it would be used as a short-term aid, between 0 and 6 months (Table 3). The initial questionnaire identified that 53% (8/15) of people felt that training required to use the app was largely suitable for the ABI population, but would need adapting to meet individual needs. Some stakeholders expressed a concern over the amount of time needed to set up, rather than learn to use BiH.

**Table 3:** Summary of questionnaire responses mapped onto BCW behaviour components

BCW component	Question/statement	Response options	Frequency	% total responses
Capability	Would you feel confident using the app? *Number scale of 1 (Definitely not) to 5 (Definitely) used.*	Definitely or almost	22/24	92%
		Maybe or somewhat	2/24	8%
	How appropriate is the app for a person with an ABI? *Number scale of 1 (Inappropriate) to 5 (Appropriate) used.*	Appropriate	6/25	24%
		Almost	11/25	44%
		Maybe	7/25	28%
		Less	1/25	4%
Motivation	When seeing the app for the first time, did it make you want to use of it? *Number scale of 1 (Definitely not) to 5 (Definitely) used.*	Definitely or almost	17/25	65%
		Maybe or less	6/25	23%
		Definitely not	2/25	8%
	What is/are the most appealing parts of the app?	Structured diary	30/41	73%
		Personalised problems and solutions	26/41	63%
		Traffic light system	22/41	54%
		Monitoring progress	15/41	37%
		Feedback online	13/41	32%
		Mentor/support	12/41	29%
	Can you see a use for the app?	Yes	39/41	95%
		No	2/41	5%
	What do you think the app could target or aid?	Memory problems	39/41	95%
		ADL	35/41	85%
		Independence	33/41	80%
		Managing routine	31/41	76%
		Problem solving	26/41	63%
		Self confidence	25/41	61%
		Anxiety/stress management	24/41	59%
		Behaviour monitoring	23/41	56%
		Anger/irritability	14/41	34%
		Goal setting	13/41	32%
		Depression	12/41	29%
Opportunity	Would you pay for the app?	Yes	9/37	24%
		Maybe	20/37	54%
		No	8/37	22%
	How long do you think you (or someone with ABI) would use the app for?	0–6 months	6/32	15%
		6–12 months	6/32	15%
		12+ months	20/32	49%
		Don’t know	9/32	22%
	How appropriate is the training (i.e., ease of learning) to use the app? *Number scale of 1 (Inappropriate) to 5 (Appropriate) used.*	Appropriate	3/15	20%
		Almost appropriate	8 /15	53%
		Maybe	3 /15	20%
		Less	1 /15	7%
	How appropriate is the app when navigating different screens? *Number scale of 1 (Inappropriate) to 5 (Appropriate) used.*	Appropriate	4/20	20%
		Almost	12/20	60%
		Maybe	4/20	20%

### Motivation

Most people said they would be motivated if they could see a use for BiH (39/41, 95%); and after physically seeing the app, 65% (17/26) of people said they wanted to use it (Table 3). The questionnaire identified that most people (39/41, 95%) felt BiH would be useful for memory problems, to help with activities of daily living (35/41, 85%) and to support independence (33/41, 80%), such as taking responsibility of appointment planning. Although users in the focus group felt BiH would be useful for self-monitoring such as fatigue or anxiety levels, only 56% (23/41) said they thought it could help with behaviour monitoring. One interesting suggestion was to use the traffic light system to monitor positive behaviours, such as a feeling of wellbeing, rather than focusing on negative behaviours. All stakeholders identified the potential for personalisation to individual needs, one of the difficulties with existing technologies, as an important enabler to uptake. ABI survivors felt that using a mobile phone app as an assistive aid was viewed as ‘normal’ behaviour, i.e., reduced stigma, and would be a motivator for use (Table 2). The main barriers identified were the lack of interest in technology, not seeing a use for it and a lack of existing evidence of its use in ABI. ABI survivors were concerned about their privacy and a feeling of being monitored, and this was expressed as a potential barrier to uptake. Stakeholders felt that setting up the app may be time consuming and excessive reminders may be annoying.

Additional points were identified by stakeholders that did not map onto the components of the BCW. Most of these related to technical aspects of the app, something that is not well reflected by the BCW model. Stakeholders highlighted the inconvenience of having to access a computer to edit the diary and functions of the app, which could be a potential barrier to uptake. If individuals have difficulty remembering events, it is important that they can add or update events easily ‘on the go’. If the system cannot be edited via the app, stakeholders felt BiH offered little additional use than a diary or the ‘reminder’ function on a standard smartphone. All stakeholders identified the need for voice recognition and the ability to verbally add things to the diary, along with having visual entries (e.g. adding pictures, diagrams and videos to diary entries, such as therapy exercises, making dinner, etc.). Professionals and carers suggested having an app specifically for mentor use, to monitor user activity without needing computer access. Professionals voiced concern over the use of the red button, the potential for it to be overused and who would be responsible for responding to it.

## Discussion

### Engagement methods

The aim of this study was to capture stakeholder views about the use of the smartphone app (BiH) in the ABI population. Both the BCW and existing technology questionnaires were used as the theoretical basis for the engagement activities. Views of ABI survivors, carers and professional were elicited through a combination of focus groups, presentations, and questionnaires. Our mixed method engagement approach provided us with useful feedback from stakeholders and enabled us to identify the importance of different opinions. The quantitative data obtained from questionnaires supported the qualitative feedback from discussions, the latter providing another dimension that would not be picked up with questionnaire responses alone. We thought the format of group engagement was helpful, as it gave stakeholders the opportunity to explore the app in more detail. Group situations promoted discussion about BiH, and stakeholders were keen to share their views about the app. The questionnaire provided useful quantifiable data, but was not completed by all.

Overall, we were able to engage well with various stakeholders, allowing us to obtain a broad range of feedback. We consulted with a stroke research group following completion of activities, who agreed that we had identified the correct stakeholders and our engagement format was appropriate. The stroke research group felt the questionnaire was suitable for the ABI population, but should be provided during focus groups or similar discussions. This would ensure stakeholders had the opportunity to explore the technology in greater depth before completing the questionnaire. Feedback from the research group reflected our findings regarding the success of focus groups, in comparison to conference presentations, where the environment did not lend itself to discussion and questionnaire completion.

### Using the behaviour change wheel model

Behaviour change techniques (BCTs), defined as ‘active components of an intervention designed to change behaviour’ [45, 46], are specific, complex components of an intervention that are designed to change behaviour, that are observable and replicable [45]. As we focused on understanding behaviours and intervention functions, rather than techniques associated with changing behaviour, we found that certain stakeholder comments did not easily map onto the BCW, e.g., the potential uses of BiH. Behaviour change interventions operate within a social context, with effectiveness being the primary criteria to consider when implementing an intervention [45]. Assessing the potential use of BiH was difficult to map onto the BCW, along with the evaluation of specific technological aspects, e.g., was it visually appealing? Therefore, the use of existing technology-specific questionnaires was necessary, alongside the BCW components and APEASE criteria.

The behaviours required to use the app were more easily mapped onto the BCW, than the potential uses. We analysed our findings by mapping enablers and barriers on to the different BCW components (Table 2). Due to the type of intervention, we were less rigid with the definitions given in the BCW, thus making it easier to categorise feedback from stakeholders. This approach was used in a similar study evaluating the use of the model [47]. We recognised that not all components of the BCW are necessary when evaluating smart technology like BiH. The BCW with the addition of enablers and barriers, appears to be a useful format that could be replicated to evaluate similar engagement activities or interventions.

### APEASE criteria

The APEASE criteria (see above) are important when evaluating interventions that aim to change behaviours, like BiH. All criteria were addressed during discussions, however stakeholders focused more on the ‘practicability’ and ‘acceptability’ aspects during focus groups, as this seemed to be most important. Practicability refers to how practical the technology would be in a real-life context and whether it is designed appropriately for the target population [34, 38, 45]. Individuals readily identified barriers and enablers that link with the practicality of use, which also mapped onto the capability and opportunity components of the BCW. Acceptability was addressed by all stakeholders via questionnaires and discussions. The acceptability of BiH differed between groups and the suggested improvements varied when speaking to ABI survivors and professionals. We were keen to ascertain whether BiH would be accepted by all stakeholders and what impact this would have on implementation. Other criteria were addressed, but some points seemed unimportant to ABI survivors and carers, such as the cost-effectiveness and unintended negative consequences of using the app. These stakeholders were interested in the potential cost of BiH for themselves or family members, but when questioned about the cost implications in the healthcare system, their feedback was minimal. As professionals did not attend focus groups, these criteria were somewhat overlooked in other engagement activities, where time and group size was a limiting factor. Therefore, we feel the BCW model needs to be fully considered prior to engaging with stakeholders and certain criteria should be prioritised depending on the group. The BCW may be used to inform qualitative interviews in further studies, which could serve as feasibility work to inform future implementation.

As previously mentioned, feedback highlighted the potential priorities for each stakeholder group. People with ABI mainly focused on the personal enablers and barriers associated with BiH uptake. They were interested in how useful and appropriate it would be for themselves, as well at the broader ABI population. This resonates with various studies, commonly stating a barrier to use is perceived usefulness and simplicity [22, 34, 48]. Professionals focused more on the clinical problems associated with using BiH (e.g. too complicated, misuse of the red button), but also the therapeutic benefits for their patients or clients. Carers were divided about their views on BiH, with some feeling it may reduce anxiety when allowing the person with ABI to have more independence, but others were concerned that it would add more pressure to their role. All stakeholders prioritised certain aspects as expected, but there was a consensus between groups regarding personalisation of the app, ability to use a smartphone, motivation to change from existing strategies. The latter has been addressed by various studies, highlighting the importance of personally relevant reminders to encourage use [6, 19, 22]. A systematic review by Ross et al., 2016 [39] presents various factors that influence the implementation of e-health technologies, like BiH. It identifies cost, complexity, adaptability, stakeholder engagement, training and education to all those involved as key factors in implementation. Our study has identified these barriers and enablers through stakeholder engagement at an early stage. We feel the data obtained appear to be a good representation of individuals that would potentially use or recommend the app.

### Limitations

The study, however, had certain limitations as the iterative approach made it difficult to plan our engagement methods before starting the study. In retrospect, we should have used the BCW to design the questionnaire alongside the existing technology questionnaires, as this would have linked better with focus group discussions. Some BCW domains were not fully explored by questionnaires and discussions e.g. policy categories and intervention functions, as we chose to focus more on the behaviour components. Questionnaires did not capture all intervention and policy areas, as it would have taken too long to complete and may be a difficult concept for some people to grasp. This meant that we did not ask stakeholders about the potential to save money within rehabilitation services, or identify potential contraindications associated with BiH use. Professionals did however highlight potential problems associated with the app. Another limitation is that stakeholders had minimal time to use and experience BiH, therefore longer term use in a case study context would provide more useful and in depth feedback.

### Future work

There is some indication that theory-based behavioural change interventions that have been led by theoretical domains framework (TDF) are more effective, than using behaviour change models alone (i.e. BCW) [49, 50]. The BCW used alongside TDF may provide better understanding of the nature of the intervention, and identify domains that could influence behaviour change [45, 50]. If we were to further this study, we could conduct single person interviews and use the TDF domain definitions to design interview questions. This approach could also be used in individual case studies.

## Conclusion

Overall people were keen the use the Brain in Hand technology, but wanted it to be personalised, easy to use and inexpensive (and potentially free). They had concerns about practical issues such as losing the phone, who had access to the data, and losing the information if the phone updated. People who currently fear or avoid using technology did not see any benefits. The majority of stakeholders said they could see the benefit of this technology for themselves or others. The BCW is a useful tool for engagement, especially when designing questionnaires and discussion topic guides. The model may help researchers consider a wider perspective than just intervention functions and associated behaviours, especially when identifying barriers and enablers to use and recommendation. However, the BCW needs additional components that are specific to AT interventions. When designing a questionnaire for this type of stakeholder engagement, researchers need to consider additional technology aspects, such as visuals, design and language. Describing our engagement process should be of interest to others who are trying to evaluate interventions designed to change behaviour.

## Additional files

Additional file 1: Engagement activity questionnaire for professionals. (DOCX 62 kb)

Additional file 2: Engagement activity questionnaire for ABI survivors and carers. (DOCX 88 kb)

## Abbreviations

ABI: Acquired brain injury
AT: Assistive technology
BCT: Behaviour change technique
BCW: Behaviour change wheel
BiH: Brain in Hand
COM-B: Capability, opportunity and motivation components of behaviour change model
HCP: Healthcare professional
MARS: Mobile Apps Rating Scale
mHealth: Mobile health
NHS: National Health Service UK
PDA: Personal digital assistant
SUS: System Usability Scale
TDF: Theoretical domains framework
