# Supplementary material for: Informing evaluation of a smartphone application for people with acquired brain injury: a stakeholder engagement study
Source: BMC Med Inform Decis Mak. 2018 May 30;18:33. doi: 10.1186/s12911-018-0611-0 (PMC5975503; doi:10.1186/s12911-018-0611-0)
Supplement: Supplementary file 1 — Engagement activity questionnaire for professionals. (DOCX 62 kb) [file 12911_2018_611_MOESM1_ESM.docx]

**Date: ______/______/______**

**1. Are you….?**  NHS Professional (please tick one box)

Private company

Other (please state) ………………………………………………………

**2. Age group:** 18-30 31-50 51+

**3. Gender:** Male Female

**4. Can you see a use for the Brain in Hand smartphone app?** Yes No

**5.** **At what stage post-injury do you think the app could be introduced?** ……………………………………………………………………………………………………………………………………………………………………………………………………………………………………………………………………………………………………………………………………………....................................

………………………………………………………………………………………………………………………

**6. How long do you think the app** 0-6 months 6-12 months 12 months+

**would be used for?**

**7. What do you think the app could target or aid?** Memory problems

(please tick all boxes that apply) Activities of daily living Problem solving

Anxiety management/stress

Depression

Goal attainment

Behaviour monitoring

Anger/irritability

Routine

Other (please state):……………………..

**8. How might Brain in Hand help your patients or clients?**

………………………………………………………………………………………………………………………

………………………………………………………………………………………………………………………

………………………………………………………………………………………………………………………

………………………………………………………………………………………………………………………

**9.** **Do you think the app is appropriate** Yes, it is appropriate

**for someone with a brain injury?**

Maybe, but needs some changes

No, it does not seem appropriate

**10. What do you think are the most appealing or** Traffic light system

**useful parts of the app?**  Mentor/support service

Structured diary

Monitoring progress

Feedback online

Personalised problem and solutions

Other (please state):………………

………………………………………...

**11. Would you be happy to pay for the app?** Yes Maybe No

**12. How do you think the app could be improved?**

………………………………………………………………………………………………………………………

………………………………………………………………………………………………………………………

………………………………………………………………………………………………………………………

………………………………………………………………………………………………………………………

………………………………………………………………………………………………………………………

………………………………………………………………………………………………………………………

**13. What would stop you from recommending the app to your patients/clients?**

………………………………………………………………………………………………………………………

………………………………………………………………………………………………………………………

………………………………………………………………………………………………………………………

………………………………………………………………………………………………………………………

**Other comments**………………………………………………………………………………………………..

………………………………………………………………………………………………………………………

………………………………………………………………………………………………………………………

………………………………………………………………………………………………………………………

………………………………………………………………………………………………………………………

………………………………………………………………………………………………………………………
