# Supplementary material for: Informing evaluation of a smartphone application for people with acquired brain injury: a stakeholder engagement study
Source: BMC Med Inform Decis Mak. 2018 May 30;18:33. doi: 10.1186/s12911-018-0611-0 (PMC5975503; doi:10.1186/s12911-018-0611-0)
Supplement: Supplementary file 2 — Engagement activity questionnaire for ABI survivors and carers. (DOCX 88 kb) [file 12911_2018_611_MOESM2_ESM.docx]

**Date: ______/______/______**

**1. Are you….?** Acquired brain injury survivor

Carer

**2. Age group:** 18-30 31-50 51+ (please tick one box)

**3. Gender:** Male Female

**4. Can you see a use for the Brain in Hand smartphone app?** Yes No

**5. What do you think Brain in Hand would be most useful for?** ……………………………………………………………………………………………………………………………………………………………………………………………………………………………………………………………………………………………………………………………………………...................................

**6. What do you think the app could target or aid?**

(please tick all boxes that apply)

Memory problems

Anxiety/stress management

Depression

Self-confidence

Behaviour monitoring

Independence

Anger/irritability

Activities of daily living

Managing routine

Problem solving

Goal setting

Other (please state) ……………………………………………………………………………………………………

**7. How could you use Brain in Hand for yourself or for someone else?**

………………………………………………………………………………………………………………………

………………………………………………………………………………………………………………………

………………………………………………………………………………………………………………………

**8. At what stage post-injury do you think Brain in Hand would be useful?**

………………………………………………………………………………………………………………………

………………………………………………………………………………………………………………………

**9. How long do you think you would** 0-6 months 6-12 months 12 months+

**use the app?**

**Think about your initial impressions of the Brain in Hand app…**

**10. Did it make you want to use it?** (please circle a number on the scale)

Not at all Definitely

1 2 3 4 5

**11. How appropriate do you think the app is for someone with a brain injury?**

(please circle a number on the scale)

Inappropriate Appropriate

1 2 3 4 5

**12. Would you feel confident using the app?** (please circle a number on the scale)

Not at all Definitely

1 2 3 4 5

**13. Would you pay for the app?** Yes Maybe No

**If yes, how much?** ……………………………………………………………………

**14. Please rate each point using the scale.** Tick one box for each question.

|  | **Inappropriate Appropriate** | | | | |
| --- | --- | --- | --- | --- | --- |
|  | **1** | **2** | **3** | **4** | **5** |
| 1. Visual (e.g. clear text, bright, stimulating) |  |  |  |  |  |
| 1. Language (e.g. easy to understand) |  |  |  |  |  |
| 1. Design (e.g. appearance, layout, colours) |  |  |  |  |  |
| 1. Using the app (e.g. clear menu) |  |  |  |  |  |
| 1. Navigating the app (e.g. accessing solutions) |  |  |  |  |  |

**15. What do you think are the most appealing or**

**useful parts of the app?** Traffic light system

Mentor/support service

Structured diary

Monitoring progress

Feedback online

Personalised problem and solutions

Other (please state):………………

………………………………………...

**17. What would stop you from using the app?**

………………………………………………………………………………………………………………………

………………………………………………………………………………………………………………………

………………………………………………………………………………………………………………………

**18. How do you think the app could be improved?**

………………………………………………………………………………………………………………………

………………………………………………………………………………………………………………………

………………………………………………………………………………………………………………………

………………………………………………………………………………………………………………………

………………………………………………………………………………………………………………………
